# From Cold to Constrained: Why MSS Colorectal Cancer Resists Immunotherapy

**DOI:** 10.66505/cbtt.v1i2.51

**Published:** 2026-05-12

**Authors:** Christophe Nicot

**Affiliations:** Department of Pathology and Laboratory Medicine, University of Kansas Medical Center, Kansas City, USA

**Keywords:** microsatellite-stable colorectal cancer, MSS colorectal cancer, immune checkpoint blockade, tumor microenvironment, immunometabolism, microbiome, immune resistance

Immune checkpoint blockade (ICB) has reshaped the treatment of multiple malignancies. However, its success in colorectal cancer (CRC) has been largely restricted to the minority of tumors exhibiting microsatellite instability-high (MSI-H) status. In contrast, microsatellite-stable (MSS) colorectal cancer remains largely refractory (1, 2). For nearly a decade, this resistance has been framed in deceptively simple terms: MSS tumors are “immune cold,” lack sufficient neoantigens, and therefore fail to respond to checkpoint inhibition. While conceptually convenient, this explanation has proven clinically insufficient. MSS CRC is not immunologically barren; rather, it is immunologically constrained. This commentary examines and contextualizes the accompanying review by Mi et al. on immune resistance in MSS colorectal cancer (3). Mi and colleagues present a timely and integrative reframing of MSS CRC immune failure, arguing that resistance to ICB is driven not by immune absence per se, but by a coordinated network of metabolic, microbial, and microenvironmental constraints that actively suppress immune competence. By integrating insights from tumor immunology, immunometabolism, and microbiome research, the authors propose a tripartite model in which host metabolism, gut dysbiosis, and immune suppression form a self-reinforcing ecosystem that limits the efficacy of checkpoint blockade alone. This reframing clarifies a long-standing misconception and directly informs therapeutic strategy. The persistence of the “cold tumor” paradigm may itself have delayed progress. By attributing failure primarily to the absence of immunogenicity, the field has often underprioritized reversible ecosystem-level barriers such as nutrient deprivation, stromal exclusion, and microbial conditioning. Reframing MSS CRC as constrained rather than inert, therefore, carries not only biological significance but therapeutic urgency.

## Beyond “cold” tumors: immune presence without immune function

A central contribution of this review is its explicit challenge to the binary “hot versus cold” tumor paradigm. Accumulating evidence indicates that many MSS CRCs harbor immune infiltrates, including CD8^+^ T cells, myeloid populations, and tertiary lymphoid structures, yet remain functionally unresponsive to ICB (4-6). This paradox underscores a key insight emphasized by Mi et al.: immune presence does not equate to immune competence (7, 8). This is not a failure of immunogenicity; it is a failure of context. The review carefully dissects how chronic antigen exposure, inhibitory receptor signaling, and metabolic insufficiency converge to drive T-cell dysfunction. Effector lymphocytes operating in glucose-depleted, hypoxic, lactate-rich environments cannot sustain cytotoxic programs, even when checkpoint signaling is relieved (9-11). In this context, checkpoint blockade in MSS CRC often releases the brake on an immune system that lacks metabolic capacity. This metabolic framing provides a mechanistic explanation for the repeated failure of ICB monotherapy despite demonstrable immune infiltration. It reframes immune resistance in MSS CRC as a problem of functional suppression rather than immune absence, with direct implications for the design and evaluation of immunotherapeutic strategies.

## Metabolism as an upstream immune checkpoint

Another major strength of the review lies in its positioning of tumor metabolism as a dominant upstream regulator of immune function. Aerobic glycolysis, hypoxia-induced HIF-1α signaling, amino acid depletion, and lipid metabolic rewiring collectively generate a tumor microenvironment (TME) that selectively favors regulatory T cells and suppressive myeloid populations over cytotoxic effectors. These metabolic constraints are not passive byproducts of tumor growth; they are actively enforced by oncogenic signaling pathways commonly operative in MSS CRC. By integrating immunometabolic principles, the authors highlight a frequently overlooked reality: immune checkpoint pathways are metabolically contingent. PD-1 signaling suppresses glycolysis and promotes fatty-acid oxidation, while hypoxia and nutrient scarcity independently inhibit mTOR- and MYC-dependent immune activation programs. As a result, metabolic stress and checkpoint signaling function as a “double brake” on antitumor immunity (12). This observation argues that metabolic reconditioning is not merely complementary to immunotherapy but may represent a prerequisite for its success in MSS CRC. These insights suggest that restoring metabolic permissiveness may be a necessary first step before checkpoint inhibition can exert meaningful clinical benefit. Checkpoint blockade cannot rescue an immune system that is metabolically incapacitated. In this framework, metabolic insufficiency is not a secondary barrier to immunity—it is a primary determinant of therapeutic failure.

## The microbiome: conditioning immune tone rather than triggering immunity

Equally compelling is the review’s nuanced treatment of the gut microbiome. Rather than portraying microbial modulation as a universal immunotherapy sensitizer, Mi et al. emphasize biological context and mechanism. Dysbiosis in CRC promotes epithelial barrier disruption, chronic inflammation, and the production of immunomodulatory metabolites, including secondary bile acids, trimethylamine-N-oxide (TMAO), and kynurenines (13). These signals collectively bias immune tone toward tolerance and suppression, reinforcing myeloid dominance and regulatory T-cell expansion. The review avoids a reductive “beneficial versus harmful bacteria” narrative. Instead, it frames the microbiome as a metabolic and inflammatory conditioning layer that interacts bidirectionally with both tumor metabolism and immune state. This framing helps explain why microbiome-targeted interventions have shown striking effects in some settings yet yielded inconsistent outcomes in CRC (14-16). The implication is clear: microbiome modulation alone is unlikely to restore immune function unless dominant metabolic and stromal constraints are addressed concurrently.

## Axis dominance as a framework for stratification

**Figure 1.**
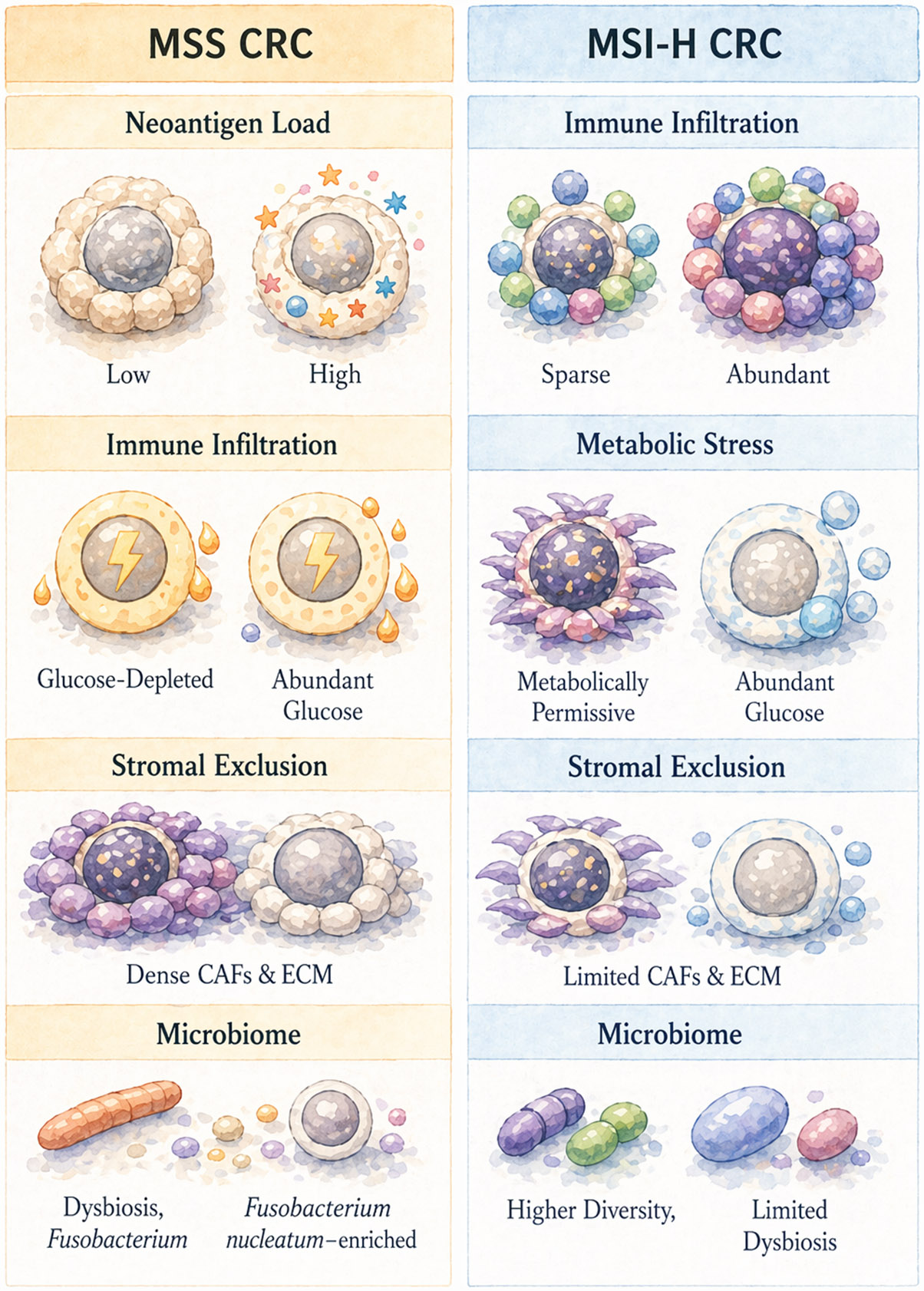
Axis-dominant immune constraint in microsatellite-stable colorectal cancer (MSS CRC). Conceptual model illustrating that immune resistance is governed by dominant suppressive axes, including metabolic stress, microbiome-driven inflammation, stromal exclusion, and immune-intrinsic checkpoint dependence. These axes impose distinct functional bottlenecks on immune activation and explain heterogeneous responses to immune checkpoint blockade, supporting the need for axis-matched therapeutic strategies. This figure was created using AI-based tools (ChatGPT/OpenAI) through iterative prompting and refinement, and was reviewed for scientific accuracy by the author.

Perhaps the most forward-looking contribution of the review by Mi and colleagues is the proposal that MSS CRC should be viewed not as a single immune-refractory entity, but as a collection of axis-dominant suppression states. Metabolically stress-dominant, microbiome-driven, stromal-exclusion, and immune-intrinsic checkpoint-dominant phenotypes impose distinct bottlenecks on immune activation (2). This framework has direct implications for trial design, arguing against empiric “add-on” approaches in favor of biomarker-guided ecosystem reconditioning. Metabolic inhibitors, microbiome modulation, stromal reprogramming, or dual-checkpoint blockade may be effective, but only when aligned with the dominant suppressive constraint in a given tumor. More broadly, it advances a decision-theoretic paradigm in which immune resistance is diagnosed before treatment and provides a unifying lens for aligning therapeutic strategies with the functional bottleneck governing immune failure in individual tumors (Figure 1).

Unlike consensus molecular subtypes or conventional immune-phenotype classifications, the axis-dominance model prioritizes identification of the actionable suppressive constraint governing immune failure in a given tumor (17). The failure of prior combination trials likely reflects axis mismatch rather than intrinsic futility (18, 19). Efforts to pair immunotherapy with metabolic or microenvironmental modulators illustrate this principle: PD-1 blockade combined with IDO inhibition failed to overcome immunosuppression when broader nutrient competition and hypoxia persisted (20). Similarly, stromal-targeting approaches, such as FAP-directed strategies, enhance immune infiltration only when stromal dominance is the primary constraint (21). Microbiome-focused interventions, including Akkermansia muciniphila enrichment, have likewise shown inconsistent efficacy in CRC despite success in other tumor types (14). Together, these observations indicate that therapeutic success depends on aligning interventions with the dominant axis of immune constraint rather than indiscriminate combinations; efficacy is conditional and diluted when only a subset of tumors depends on the targeted axis.

## Toward ecosystem reconditioning as a therapeutic paradigm

**Figure 2.**
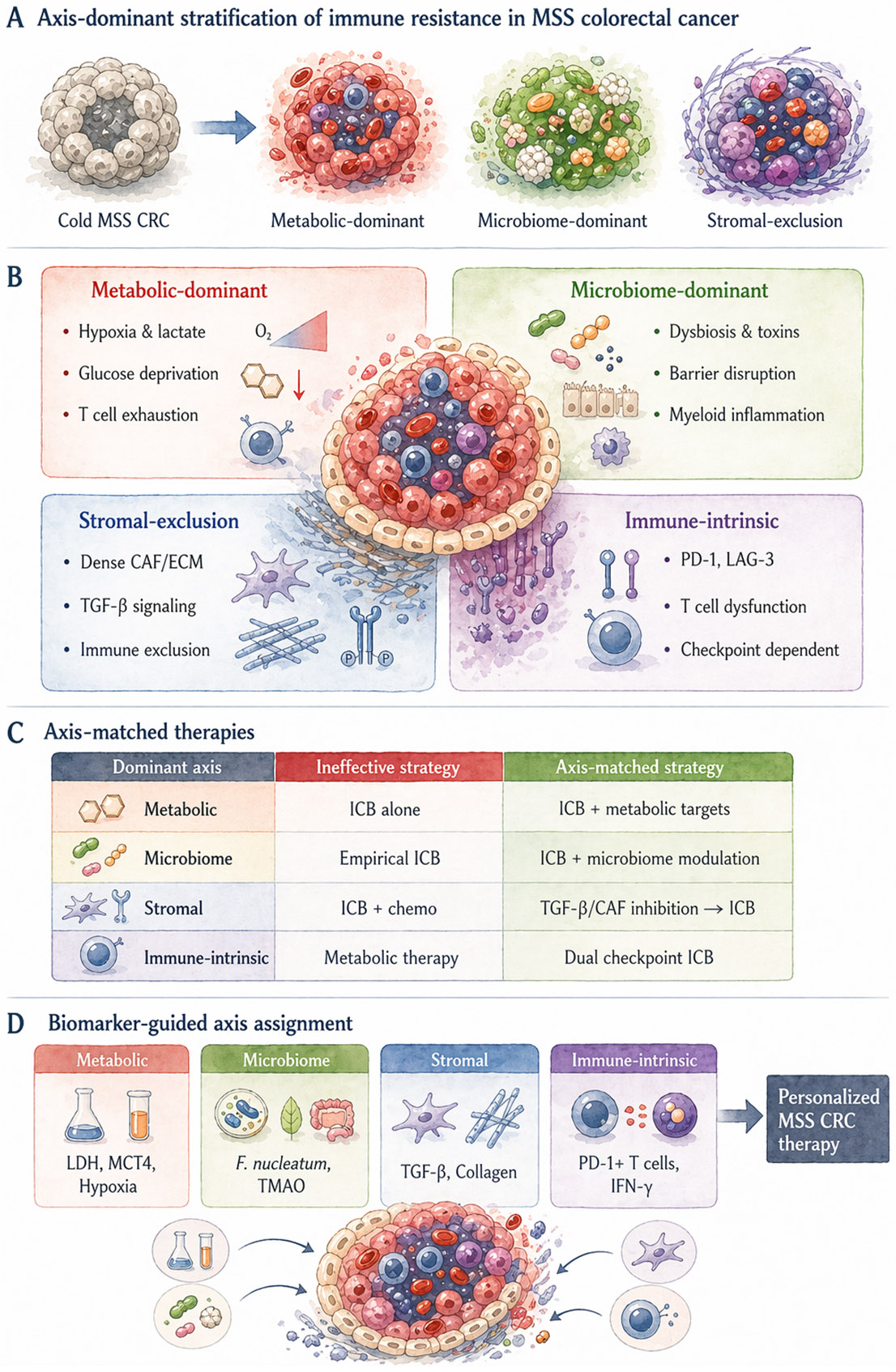
Immune constraint landscapes in microsatellite-stable versus microsatellite instability-high colorectal cancer. Conceptual schematic comparing determinants of immune responsiveness in MSS and MSI-H colorectal cancer. MSS tumors are characterized by metabolically restrictive microenvironments, stromal exclusion, and microbiome dysbiosis, which constrain T-cell function despite the presence of immune cells. In contrast, MSI-H tumors exhibit high neoantigen burden, functional immune infiltration, reduced stromal barriers, and a more permissive immune context. This comparison highlights that immune responsiveness is governed primarily by interactions within the tumor ecosystem rather than by tumor cell identity alone. This figure was created using AI-based tools (ChatGPT/OpenAI) through iterative prompting and refinement, and was reviewed for scientific accuracy by the author.

Immune resistance in MSS CRC is a dynamic, multilayered state shaped by metabolic stress, hypoxia, suppressive metabolites, microbial signals, and stromal architecture. Releasing inhibitory receptors without addressing these upstream constraints is unlikely to produce a durable benefit (Figure 2).

The next generation of MSS CRC immunotherapy trials should not be judged solely by response rate. Early metabolic normalization, stromal remodeling, restoration of T-cell fitness, or microbiome reprogramming may represent meaningful intermediate endpoints that precede objective tumor regression. The translational implications of the axis-dominance framework are outlined in Table 1, which links dominant suppressive constraints in MSS colorectal cancer with candidate biomarkers and rational therapeutic strategies.

Integration of spatial transcriptomics, metabolomics, and microbiome profiling will be essential to operationalize this framework (22, 23). Such approaches can reveal dominant suppressive axes within individual tumors and guide rational combination strategies. While challenges remain, including biomarker validation, therapeutic toxicity, and clinical feasibility, the ecosystem-reconditioning paradigm offers a coherent path beyond the stagnation that has characterized MSS CRC immunotherapy to date.

By reframing MSS CRC as constrained rather than cold, this review provides both conceptual clarity and practical direction. It invites the field to move beyond reductive explanations toward a more precise, systems-level understanding of immune resistance, one that may ultimately enable durable immunotherapeutic benefit for the majority of patients with colorectal cancer. The field now faces a clear mandate: diagnose the dominant axis of immune constraint in each tumor and intervene accordingly.

MSS colorectal cancer is not immune “cold” but functionally constrained by metabolic, microbial, and microenvironmental barriers. This reframes resistance as a systems-level problem, positioning metabolic and ecological constraints as primary determinants of response. Progress will depend not on intensifying checkpoint blockade, but on diagnosing and dismantling the dominant constraint in each tumor. This shift from immune activation to ecosystem reconditioning represents a necessary evolution for achieving durable immunotherapy responses.

## Figures and Tables

**Table 1.** Dominant immune-constraint axes in microsatellite-stable colorectal cancer: candidate biomarkers and rational therapeutic strategies. **Abbreviations:** HIF1α, hypoxia-inducible factor 1α; LDHA, lactate dehydrogenase A; CAF, cancer-associated fibroblasts; FAP, fibroblast activation protein; FMT, fecal microbiota transplantation; MDSC, myeloid-derived suppressor cells; TAM, tumor-associated macrophages; PD-1, programmed cell death protein 1.

Dominant Axis	Representative Biomarkers	Functional Effect	Potential Therapeutic Strategy
Metabolic stress	HIF1α, LDHA, lactate signature	T-cell dysfunction	Metabolic inhibitor + PD-1
Stromal exclusion	TGF-β, CAF/FAP signature	Immune exclusion	Stromal/TGF-β targeting
Microbiome dysbiosis	Microbial composition, bile acid profile	Tolerogenic inflammation	Microbiome modulation / FMT
Checkpoint dominant	PD-L1, exhausted T-cell markers	Adaptive inhibition	Dual checkpoint blockade
Myeloid suppression	MDSC/TAM signatures	Antigen suppression	CSF1R / myeloid targeting

## Data Availability

“No datasets were generated or analyzed in the current study.”
